# Immune Checkpoint Inhibitor-Induced Hemorrhagic Gastritis

**DOI:** 10.14309/crj.0000000000001128

**Published:** 2023-08-14

**Authors:** Karthik Mathialagan, Cheng-Hung Tai, Samdish Sethi, Sumi Thomas, Caroline Loeser

**Affiliations:** 1 Department of Medicine, Yale-New Haven Health–Bridgeport Hospital, Bridgeport, CT; 2 Section of Gastroenterology, Department of Medicine, Yale-New Haven Health–Bridgeport Hospital, Bridgeport, CT; 3 Department of Pathology, Yale University School of Medicine, New Haven, CT

**Keywords:** hemorrhagic gastritis, pembrolizumab, immune checkpoint inhibitor, immune related adverse events

## Abstract

Immune checkpoint inhibitors (ICIs) have been increasingly used in the treatment of several malignancies and may target cytotoxic T-lymphocyte-associated antigen-4, programmed cell death-1, and programmed cell death ligand 1, which work on maintaining peripheral immune tolerance. ICIs inhibit these ligands causing an immune-enhancing effect, leading to a wide spectrum of complications from mild mucositis to life-threatening pneumonitis or hepatitis. These complications are collectively called immune-related adverse events. Their prevalence has increased with a rise in ICI use, with rare manifestations being reported in popular literature. We present a case of hemorrhagic gastritis due to the anti-programmed cell death-1 antibody, pembrolizumab.

## INTRODUCTION

Immune checkpoint inhibitors (ICIs) work through specific targets to enhance the immune system and prime them to attack cancer cells.^1^ Despite known benefits, this mechanism also causes an autoimmune response that affects several organs classified as ICI-mediated immune-related adverse events (irAEs). It encompasses a wide clinical spectrum ranging from mild colitis to life-threatening pneumonitis. Increasing its recognition would enable providers to allow for earlier detection and effective management.^1^ In this article, we report a rare complication of hemorrhagic gastritis due to pembrolizumab, which targets programmed cell death 1 (PD 1).

## CASE REPORT

**Figure 1.**
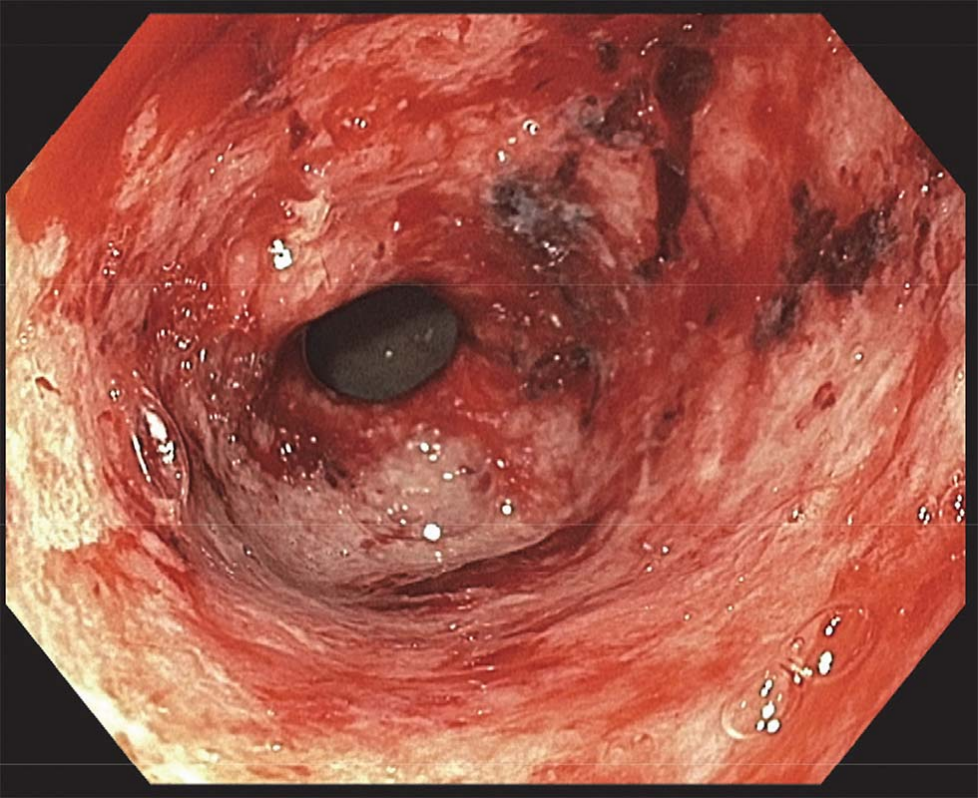
Upper endoscopy showing diffuse hemorrhagic gastric mucosa with bleeding.

**Figure 2.**
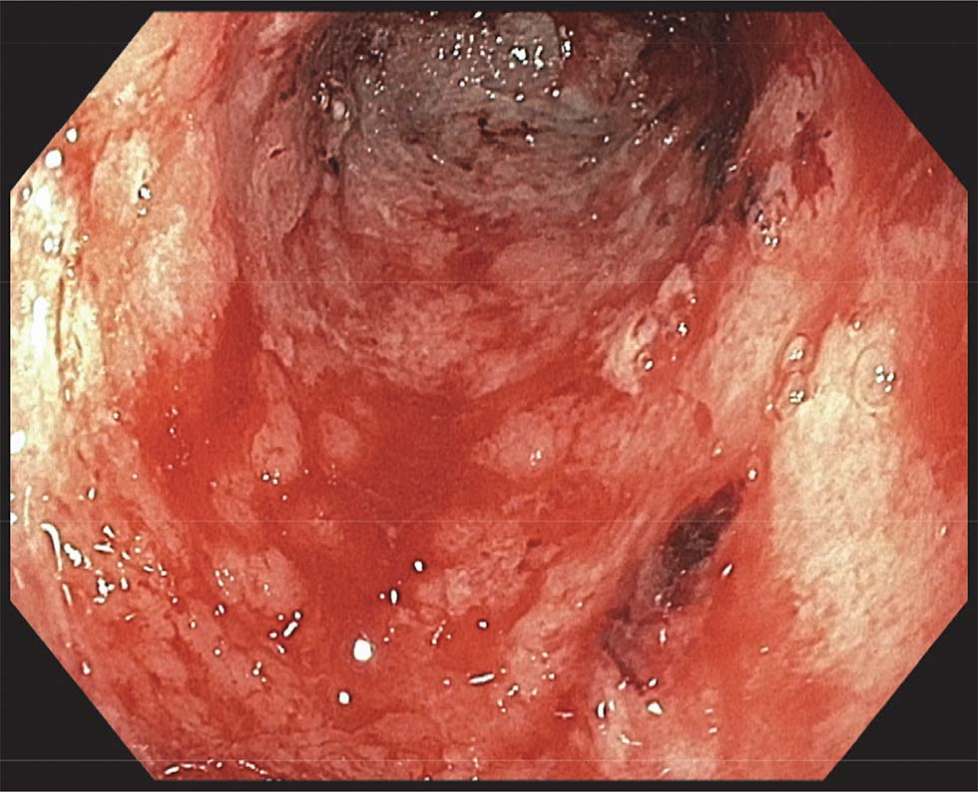
Upper endoscopy showing diffuse hemorrhagic gastric mucosa with bleeding.

**Figure 3.**
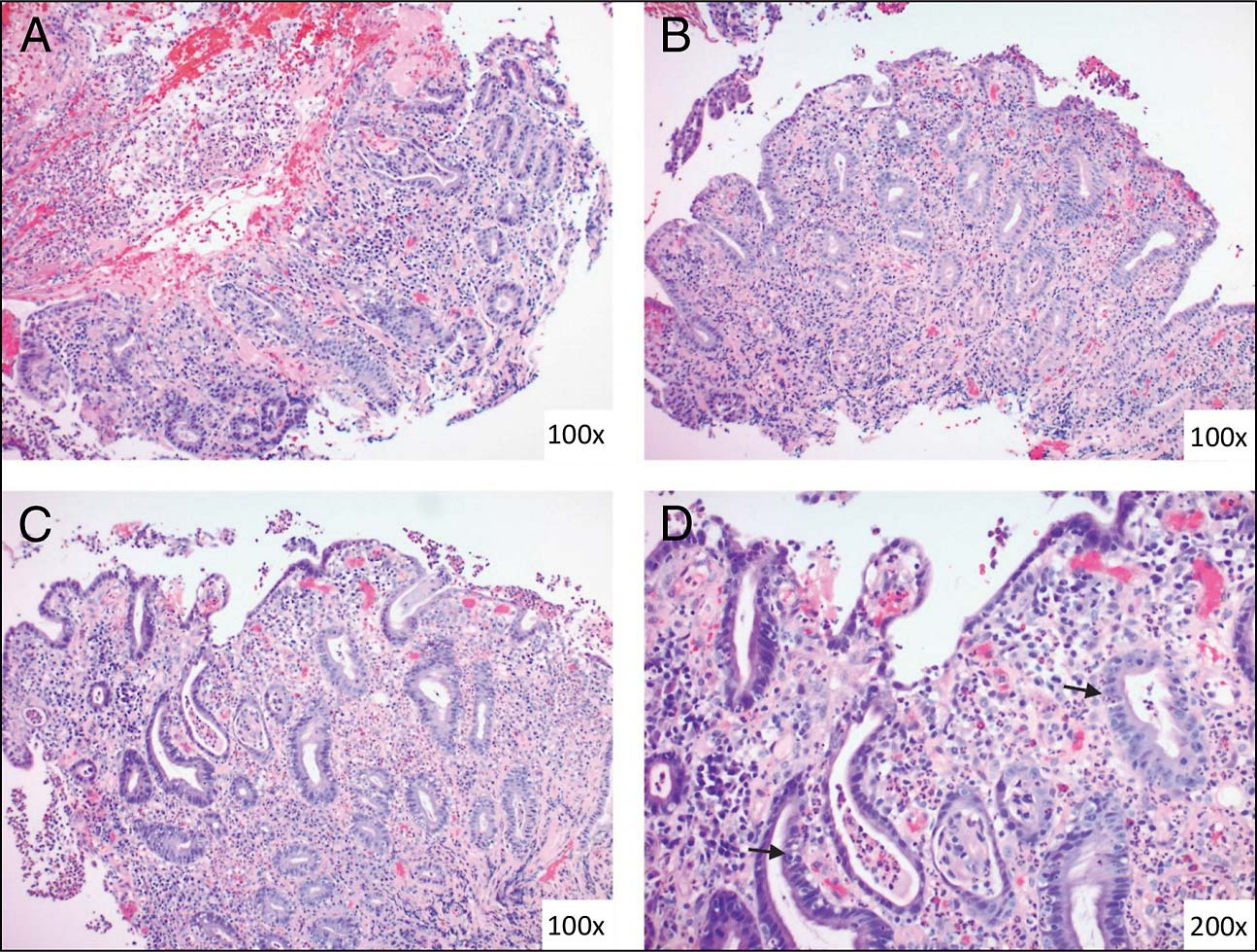
Gastric biopsies showing severe acute gastritis. (A) Ulceration and fibrinopurulent material (100×). (B and C) Lamina propria infiltration by neutrophils, plasma cells, and eosinophils (100×). (D) Intraepithelial neutrophils and lymphocytes (200×).

A 76-year-old woman presented with hematemesis. Her medical history was notable for stage 4B uterine carcinosarcoma for which she was initially treated with carboplatin and paclitaxel, followed by pembrolizumab. Pembrolizumab was briefly discontinued for suspicion of ICI hepatitis. She was then found to have tumor progression a year later and was reinitiated on pembrolizumab. While on pembrolizumab, the patient developed nausea, hematemesis, and melena. Initial vitals were within normal limits. Blood work was notable for blood urea nitrogen 8 mg/dL, creatinine 0.56 mg/dL, and hemoglobin 8.1 g/dL and was otherwise unremarkable. Upper endoscopy revealed diffuse hemorrhagic gastritis with bleeding in the gastric antrum (Figures 1 and 2). Histopathology was notable for severe acute gastritis with mucosal erosion, ulceration, and neutrophilic abscesses. The glands showed intraepithelial neutrophils and lymphocytes. The lamina propria had a mixed inflammatory infiltrate of neutrophils (predominant), plasma cells, lymphocytes, and eosinophils (Figure 3). Immunostains were negative for *Helicobacter pylori*, cytomegalovirus, and herpes simplex virus. Given the history of pembrolizumab use, biopsy findings were found to be consistent with ICI-induced gastritis.^2^ Pembrolizumab was discontinued, and she was discharged on high-dose prednisone taper. Repeat upper endoscopy 8 weeks later revealed complete resolution of mucosal changes with only localized minimal inflammation seen in the gastric antrum.

## DISCUSSION

Hemorrhagic gastropathy, used to describe gastric mucosal bleeding without ulcerative disease, is most frequently observed in association with nonsteroidal anti-inflammatory drugs, alcohol use disorder, and critical illness. Other causes that lead to gastric mucosal inflammation include viral gastritis, *H. pylori*, and celiac disease.^3^ This patient presented with no such risk factors or prior exposure to anticoagulant medications. Biopsy confirmed the absence of herpes simplex virus, cytomegalovirus, and *H. pylori* and demonstrated a pattern of histopathology consistent with ICI-mediated gastritis.^4–6^ ICIs, such as pembrolizumab, are known to cause irAEs, such as colitis and hepatitis.^7^ ICI-mediated inflammatory changes have been well-described in the colonic mucosa and liver, but less so in the gastric mucosa.^2^ A study comparing histopathological changes between *H. pylori*, celiac disease, and ICI-mediated inflammation on the upper gastrointestinal (GI) tract was performed to delineate the subtle differences in their individual morphology.^2^ The different characteristics of ICI-mediated gastroduodenitis include less marked lamina propria inflammation, numerous intraepithelial lymphocytes because of a higher level of intraepithelial CD8 T cells, fewer lymphoid aggregates, and increased neutrophilic infiltrate and erosions.^2^ Although ICI-mediated gastritis has been reported in the literature, hemorrhagic complication due to this entity is extremely rare because it is seen in only a handful of cases.^4–6,8,9^

IrAEs related to ICIs are typically organ-specific, and onset in some cases may be delayed by months.^10^ IrAEs related to anti-PD-1 antibodies (such as nivolumab and pembrolizumab) differ in frequency and organ involvement compared with other ICIs, and observed complications may be more severe.^11^ Lower GI complications occur more frequently than upper GI complications.^12^ A review of the current literature revealed gastritis related to irAEs was higher with ICI monotherapy, with 14 of 36 cases of severe gastritis occurring in the setting of pembrolizumab monotherapy, of which only 2 cases were associated with hemorrhagic gastritis.^13^ We reviewed 6 other case reports with ICI-induced hemorrhagic gastritis, of which 3 were associated with pembrolizumab monotherapy, 1 with nivolumab monotherapy, 1 with ipilimumab monotherapy, and 1 with ipilimumab and nivolumab combination therapy.^4–6,8,9,14^

ICIs act on the modulation of T-cell response to the antigen. After activation of T cells through antigen recognition and costimulation, PD-1 expression is induced.^15^ Activation through PD-1 leads to inhibition of peripheral T cells, B cells, macrophages, and dendritic cells through apoptosis and other regulatory mechanisms. Thus, anti-PD-1 antibodies have an immune-enhancing effect causing a proinflammatory state and autoimmunity.^15^ Current guidelines recommend holding ICI for grade 3 toxicities and initiating systemic steroids at the dose of 1-2 mg/kg/day, tapering over the course of 4-6 weeks. If symptoms do not improve over 72 hours, treatment with infliximab may be considered. In the study by Sugiyama et al, most of the patients improved on systemic steroids while infliximab was required in 2 of these 36 cases of severe gastritis. Similarly, infliximab was used in 1 patient with hemorrhagic gastritis reported by Cinnor.^4,13^ Ongoing studies centered around unveiling the morphological and immunological distinction between irAEs and other conditions that affect the upper GI tract may help clinicians better identify irAEs, although further studies are needed to understand the molecular mechanisms of its immune dysregulation.

## DISCLOSURES

Author contributions: K. Mathialagan performed initial chart review and review of literature to produce the initial manuscript draft and is the article guarantor. C. Tai conducted revision of the manuscript and led content designing and literature review. S. Thomas was responsible for microscopy slides. S Sethi led additional review of the literature and referencing. C. Loeser was the supervising clinician and edited the final manuscript.

Financial disclosure: None to report.

Informed consent was obtained for this case report.
